# Sheaving—a universal construction for semantic compositionality

**DOI:** 10.1098/rstb.2019.0303

**Published:** 2019-12-16

**Authors:** Steven Phillips

**Affiliations:** National Institute of Advanced Industrial Science and Technology, Tsukuba 305-8566, Japan

**Keywords:** compositionality, category theory, sheaf, sheaving, topology

## Abstract

Semantic compositionality—the way that meanings of complex entities obtain from meanings of constituent entities and their structural relations—is supposed to explain certain concomitant cognitive capacities, such as systematicity. Yet, cognitive scientists are divided on mechanisms for compositionality: e.g. a language of thought on one side versus a geometry of thought on the other. Category theory is a field of (meta)mathematics invented to bridge formal divides. We focus on sheaving—a construction at the nexus of algebra and geometry/topology, alluding to an integrative view, to sketch out a category theory perspective on the semantics of compositionality. Sheaving is a universal construction for making inferences from local knowledge, where meaning is grounded by the underlying topological space. Three examples illustrate how topology conveys meaning, in terms of the inclusion relations between the open sets that constitute the space, though the topology is not regarded as the only source of semantic information. In this sense, category (sheaf) theory provides a general framework for semantic compositionality.

This article is part of the theme issue ‘Towards mechanistic models of meaning composition’.

## 1. Introduction

The way that representations and their meanings for complex entities obtain from the representations and meanings for the constituent entities and their structural relations is called *semantic compositionality*. Some form of compositionality is supposed to explain concomitant cognitive capacities, such as the *systematicity* of language [1] and thought [2], i.e. where possessing certain cognitive capacities implies possessing certain other (structurally related) cognitive capacities—an equivalence relation on cognitive abilities [3]—such as understanding the meaning of *John loves Mary* and *Mary loves John*. *Classical compositionality*, for example, supposedly explains systematicity by appealing to a *combinatorial syntax and semantics*, i.e. semantic relations between constituent entities are captured by syntactic relations between corresponding symbols, and cognitive processes that are sensitive to those syntactic/semantic relations [4]. So, to illustrate, the meaning of *John loves Mary* is captured by a symbol, JOHN, for the person *John*, a symbol, MARY, for the person *Mary*, and a symbol, LOVES, for the relation *loves* that are combined (juxtaposed) in a way that expresses the fact that *John* is the lover and *Mary* is the one loved, e.g. LOVES (JOHN, MARY). Classical compositionality can account for systematicity by assuming that structurally related capacities involve the same combinatorial syntactic process [4], e.g. lover is determined by the first argument position. Analogous accounts are supposed for other (non-classical) forms of compositionality, e.g. as used in connectionist (neural network) models, where symbols are replaced with vectors of neural activity and juxtapositioning with operations such as tensor product [5]. Though the claim that these forms are non-classical has been extensively debated [6], they generally suppose that semantic relations are captured by relations between corresponding representations.

Despite the efficacy of supposing a compositionality principle, cognitive scientists are generally divided on symbolic [4] versus non-symbolic [5] mechanisms—a *language of thought* [2] on one side versus a *geometry of thought* [7] on the other—and their explanatory import [6]. The challenge is not just to explain how some form of compositionality accounts for properties such as systematicity, but *why* cognition is compositional in the first place [8].

Explaining the *why* versus *how* of systematicity was posed as a challenging problem for connectionist theories [4], and later shown to be also problematic for classical theory [6]. Problematically, while there are instances of compositionality that support a requisite systematicity property, there are also instances that do not support the same property. So, systematicity does not necessarily follow from core principles and assumptions of classical or connectionist theories. Auxiliary assumptions added to pick out just those instances of compositionality that support systematicity are *ad hoc* when they are unconnected to the theory’s core principles and assumptions, cannot be confirmed independently of confirming the theory, and are motivated only by the need to fit the data, in which case, the theory fails to fully explain systematicity [6]. One recourse is to claim that the supposed counterexamples are not the ‘canonical’ forms of compositionality that classical theory takes as a core assumption [3]. Yet, its unclear what characterizes canonicity, or why cognition is canonically compositional [9].

A *category theory* [10] approach to compositionality was introduced to address the *why* of systematicity [11]. Category theory is a field of (meta)mathematics invented to formally compare mathematical structures [12]. The core explanatory concept is *universal construction*, formalized as *universal morphism*, which is a way of comparing cognitive capacities modelled as compositions of maps—such constructions are characterized by a *universal mapping property* [13]: in regard to a collection of systematically related cognitive capacities, each map modelling a member capacity is composed of the map shared by all members and a map that is unique to that capacity. Hence, a universal morphism identifies an equivalence class of systematically related cognitive capacities. Such constructions are the ‘best’ one can do within a certain (categorical) context—every construction in that context ‘leads to’ a universal construction, so necessarily obtains via a recursive process [9].

An explanation for semantic compositionality must ultimately connect to the physical (neural) system that supports cognition. Classical theory assumes that symbols are supported by a neural system that implements the equivalent of memory registers, i.e. the *physical symbol system* hypothesis [14]. Connectionist theory makes this link more directly as the representations that supposedly support semantic compositionality are instantiated as neural activity for a network of (abstract) neurons. A categorical approach must also make this kind of connection. To this end, the current work focuses on another universal morphism, called *sheaving* [15] or *sheafification* [16], to sketch out a category theory perspective on the semantics of compositionality. Sheaving is a construction at the nexus of algebra and geometry/topology, which alludes to an integrative view. This view starts with a *(pre)sheaf* to model cognitive representations as data attached to a topological space [17]. As we shall see, the underlying topological space gives meaning to the data in terms of the relations between the open sets that constitute the topology.

The presentation of this work is primarily informal to facilitate an intuitive understanding of the approach. Connections to formal details appear elsewhere [17], and deeper introductions to categories and sheaves appear in many textbooks on these topics [10,16,18,19]. We proceed with an example of a universal morphism that serves to illustrate the basic category theory concepts (§2) underlying the examples of sheaving given in the context of cognition (§3). This approach is discussed by comparison and contrast with classical notions of compositionality and possible neural mechanisms (§4). For convenience and to help ground concepts, some formal details appear in the appendix.

## 2. Categories and (universal) compositionality

We use *playing cards* as a running example of compositionality to bootstrap the needed category theory from the more familiar concepts of sets and functions. Each card has a *rank* (i.e. *two*, *three*, … , *ten*, *jack*, *queen*, *king*, *ace*) and a *suit* (i.e. *spade*, *club*, *diamond*, *heart*). For example, *queen* and *heart* constitute the *queen of hearts*. The ranks can be represented by the set of symbols Rank={2,3,4,5,6,7,8,9,10,J,Q,K,A}, the suits by the set of symbols Suit={♠,♣,♢,♡} and the cards by the *Cartesian product* of those sets: Card=Rank×Suit={(2,♠),(2,♣),…}. For instance, the pair of symbols (Q,♡) represents the *queen of hearts*. This product also comes with two functions that retrieve the rank and suit of each card: e.g. rk : (Q,♡)↦Q and st : (Q,♡)↦♡. Accordingly, sets and functions provide a basic set-theoretic model of *playing cards*.

Category theory starts with the formal concept of a *category* (definition A.1), which consists of a collection of entities, called *objects*, a collection of relations between objects, called *morphisms*, and an operation that takes two morphisms and returns a morphism, called *composition*. The archetypal category is **Set** (example A.2), the category of sets (objects) and functions (morphisms), with function composition as the composition operation (remark A.3). Hence, sets *Rank*, *Suit* and *Card* are objects and functions *rk* and *st* are morphisms in **Set**, constituting a *categorical product* (definition A.6), which is the Cartesian product for this category (example A.7). A deck of cards is modelled as a mapping of each face, signifying a *playing card*, to the corresponding symbol, e.g. a function $card\;:\;Face\to Card;Q^{♡}\mapsto (\text{Q},♡)$. The mappings from faces to ranks and from faces to suits are given by compositions  faceRank=rk∘card and  faceSuit=st∘card, respectively: e.g. $\;faceRank\;:\;Q^{♡}\mapsto \text{Q}$, which says that the rank of the card signified by the face $Q^{♡}$ is Q (remark A.8). Thus, we have a category-theoretic model of the same *playing cards* concept.

Having introduced categories, we can now look at basic constructions and their relations. A *functor* (definition A.12) is a way of constructing, indexing, or identifying objects and morphisms. For example, the product functor (example A.14) constructs the set of cards from the sets of ranks and suits, i.e. Π : (Rank,Suit)↦Rank×Suit, and a constant functor identifies the set of cards (i.e. the functor that sends every set and function, in **Set**, to the set of cards, Card, and its identity function, $1_{Card}$). Two functors are related by a *natural transformation* (definition A.15), and the optimal (or most efficient) transformation pertains to a *universal morphism* (definition A.17). For example, the transformation from the set of cards to their ranks and suits is the universal morphism (Card,rs), where rs={rk,st}. The transformation is efficient in that there are no more and no fewer mappings than needed to retrieve the rank and suit of every card.

Note that universal morphisms are *unique up to unique isomorphism* (remark A.19). So, constituents need not be ‘tokened’ in the classical sense. A characteristic of classical compositionality is that the symbols representing constituents are *tokened* (inscribed, or written out) whenever the representation of their complex host is tokened [4]. The symbol pair representation of cards is an example of tokening: for instance, the symbols for *queen*, Q, and *heart*, ♡, are tokened whenever the symbol for *queen of hearts*, (Q,♡), is tokened. In category theory, the product of two sets is conventionally given as the Cartesian product, but other products exist. For example, the cards can be represented as numbers, say from 1 to 52, provided the accompanying functions retrieve the requisite components. Being an isomorphic set is not sufficient, because one still needs the appropriate functions to recover the constituents—such isomorphisms are generally not unique (remark A.19).

## 3. Sheaving: bridging gaps in knowledge

Our categorical approach to semantic compositionality involves *presheaves/sheaves* (functors) and *sheaving* (natural transformation). A presheaf/sheaf (definitions A.20/A.21) models data attached to a topological space (definition A.4). A sheaf is a presheaf where the attached data are globally coherent, i.e. agree on overlapping regions. *Pullbacks* (definition A.9) express global coherency conditions (remark A.22). For **Set**, a pullback of *f* and *g* (example A.10) is a constrained product (remark A.11), which consists of only those pairs, (*a*, *b*), whose components map to a common value (property): *f*(*a*) = *g*(*b*). Hence, pullbacks pertain to non-local (global) properties. Sheaving is a universal morphism that constructs the ‘nearest’ sheaf from a given presheaf (remark A.23). This construction is likened to the *natural join* operation (example A.24) that extracts information from data stored locally in different tables of a relational database—say, the addresses of all people prescribed a particular medication, where contact and medical data are stored in separate tables. In this way, sheaving is a kind of relational inference: a way of bridging gaps in knowledge via meaning grounded in the underlying topological space.

We give three examples of sheaving that pertain to cognition. The first example continues the introduction to category (sheaf) theory constructions via the familiar concept of *playing cards*. The second example involves visual feature binding [17] extended for triple conjunction search [20]. The third example involves a simple version of depth perception. Each example illustrates the different ways that meaning is conveyed by the relations between the open sets that constitute the topology.

### (a) Playing cards

The *playing cards* example, introduced earlier, can be considered as a presheaf or sheaf on a topological space constituted by elements identifying the (feature) dimensions of rank and suit. For example, suppose the rank and suit dimensions are labelled as R and S, respectively. The set of dimension labels *D* = {R, S} together with the topology {∅,{R},{S},{R,S}} constitute a *discrete topological space*, which consists of all subsets of labels and their inclusion relations (example A.5). And, the values of each card constitute the data attached to that space. For example, the *queen of hearts* and *two of spades* are represented by the presheaf, $F_{Q2}\;:\;D^{\mathrm{op}}\to Set$. In database terms, this presheaf can be regarded as a collection of tables whose attributes (headings) correspond to the open sets and rows correspond to the attached data, e.g. there is a two-column table whose attributes correspond to the open set {R, S} that has two rows: one row for the *queen of hearts* and one row for the *two of spades* (example A.26). In sheaf theory terms, $F_{Q2}$ sends each open set to the set of functions on that set—each function maps the elements of the open set to the attached data—e.g. $F_{Q2}\;:\;\{\text{R},\text{S}\}\mapsto \{c_{\mathrm{QH}},c_{2S}\}$, where $c_{\mathrm{QH}}\;:\;\text{R}\mapsto \text{Q},\text{S}\mapsto ♡$ and $c_{2S}\;:\;\text{R}\mapsto 2,\text{S}\mapsto ♠$. The inclusions given by the topology are preserved as restrictions on functions, e.g. {R} ⊆ {R, S} maps to the restriction $f{|}_{R}\;:\;c_{\mathrm{QH}}\mapsto c_{Q},c_{2S}\mapsto c_{2}$. Restriction corresponds to (database) projection of a table along the specified attribute(s).

Sheaving affords the systematic capacity to represent all cards (example A.27), but this capacity depends on the topology. To illustrate, suppose one knows the ranks and suits, i.e. there is a one-column table of 13 rows for ranks and a one-column table of four rows for suits. In this situation, sheaving simply constructs all pairwise combinations of ranks and suits, which is the sheaf $F_{\mathrm{card}}^{+}$. Thus, we have a systematic capacity to represent all 52 cards. One can think of sheaving as a kind of completion, or limit process—adding just enough rows to make a sheaf.

A contrasting scenario is where one knows some of the cards without knowing about constituents *rank* and *suit*: cards are understood as non-compositional entities. This situation is captured by the *indiscrete topology* (example A.5), i.e. {∅,D}. Sheaving, in this case, does not add any rows to the table containing just the known (non-compositional) cards. Hence, one does not necessarily have a systematic capacity to represent all cards. Completion is trivial—the presheaf is a sheaf—because the topology does not consist of any other (non-empty) open sets.

This difference between sheaving with respect to a discrete versus indiscrete topological space was used to model the difference between generalization and lack of generalization observed with participants trained on cue-target maps [17]. The participants who failed to generalize were regarded as having learned the mappings from cues to targets—pairs of letters to coloured shapes—as mappings of non-compositional entities.

### (b) Visual feature binding

Visual feature binding concerns the capacity to identify, say, a red square and a blue triangle, as opposed to a red triangle and a blue square based on globally coherent spatial information (location). This process is modelled as the sheaving of colour and shape location maps to obtain a colour-shape conjunction map that corresponds to objects observed in the visual field as needed to perform visual search [17]. Here, we show how this example of sheaving extends straightforwardly to triple conjunction search [20], i.e. where the target of search is identifiable by a triple of features, such as colour, orientation and (spatial) frequency.

In this example, we start with the set of dimension labels *D* = {C, O, F, L} representing colour, orientation, frequency and location. The feature-location maps are data attached to the corresponding open sets of the topology: {∅,{L},{C,L},{O,L},{F,L},{C,O,L},{C,F,L},{O,F,L},{C,O,F,L}}. For instance, suppose there are four locations, i.e. *L* = {1, 2, 3, 4}, two colours C={red,blue}, two orientations O={hor,ver} and two frequencies F={low,high}. In database terms, the identification of the four objects, indexed by location, (*red, hor, low*)_1_, (*red, hor, high*)_2_, (*blue, hor, low*)_3_, (*red, ver, low*)_4_ obtains from the natural join of the corresponding feature-location tables: e.g. the join of the colour-location table with rows (*red*)_1_, (*red*)_2_, (*blue*)_3_, (*red*)_4_ and the orientation-location table with rows (*hor*)_1_, (*hor*)_2_, (*hor*)_3_, (*ver*)_4_ obtains the colour-orientation table with rows (*red, hor*)_1_, (*red, hor*)_2_, (*blue, hor*)_3_, (*red, ver*)_4_; and the join of colour-orientation with frequency-location (*low*)_1_, (*high*)_2_, (*low*)_3_, (*low*)_4_ obtains the four objects.

In terms of universal morphisms, sheaving involves pullbacks (remark A.22). For instance, the colour-orientation map obtains from the pullback of the projections of the colour-location (CL) and orientation (OL) maps onto location: $\pi_{2}\;:\;CL\to L$ and $\pi_{2}\;:\;OL\to L$ to obtain the colour-orientation map, denoted *C* ×_*L*_ *O*, and its projections. Thus, triple conjunction obtains from two pullbacks: (*C* ×_*L*_ *O*) ×_*L*_ *F*.

The topology in this example conveys a different (relational) meaning from the meanings conveyed by the discrete and indiscrete topologies. Each topology induces a corresponding order over the elements of the underlying space, called the *specialization (pre)order* (remark A.28): C ≤ L, O ≤ L, F ≤ L for the current example, which says that colour, orientation and frequency specialize location; conversely, location is a general (global) property of the data (object features) attached to the topological space. By contrast, the discrete topology in the cards example has the corresponding order R ≤ R, S ≤ S, which says that neither dimension is a specialization of the other. In other words, the dimensions are independent; sheaving is effectively a Cartesian product of the sets of values on those dimensions (example A.27). The preorder corresponding to the indiscete topology in the cards example has R ≤ S and S ≤ R, which says that the dimensions are specializations of each other, i.e. effectively the same dimension (remark A.28). Thus, topology plays a significant role in our approach to semantic compositionality.

### (c) Depth perception

Binocular vision can be used to infer (triangulate) location of a target object using lines of sight and relative eye positions. This computation can be achieved as an instance of sheaving, using simple geometry. Suppose the position of the target object is (*x*, *y*) ∈ *P* and the angles of the eyes (lines of sight) to the target are *λ* and *ρ* for the left and right eyes, respectively. Left and right lines of sight specify position as functions of distance from the eyes, *l* ∈ *L* and *r* ∈ *R*, parameterized by angle:

— ${left}_{\lambda }\;:\;l\mapsto l(\cos\lambda ,\sin\lambda )$, and
— ${right}_{\rho }\;:\;r\mapsto r(\cos\rho ,\sin\rho )$.

The position of the target is the intersection of the two lines of sight, which is the pullback of ${left}_{\lambda }$ and ${right}_{\rho }$. This pullback is equivalent to the pullback of projections $\pi_{2}\;:\;LP\to P$ and $\pi_{2}\;:\;RP\to P$, where LP is the relation $\left\{(l,{left}_{\lambda }(l))|l\in L\right\}$ and RP is the relation $\left\{(r,{right}_{\rho }(r))|r\in R\right\}$. Suppose the set of distance and position labels {L, R, P} is given the topology {∅,{P},{L,P},{R,P},{L,R,P}}. This topology is analogous to the previous (binding) example, so it conveys similar meaning. Sheaving on this space computes the pullback of the projections (remark A.22). Hence, the location of the target object is obtained as an instance of sheaving.

## 4. Discussion

Semantic compositionality concerns the way that representations and the entities they stand in for correspond in some systematic, structurally consistent manner. Our sheaf theory approach regards this correspondence as data attached to a topological space (presheaf/sheaf), where the shape (topology) of the underlying space conveys meaning to the representations. Shape is determined by the open sets and its structure is preserved by restrictions of the data, either locally (presheaf), or in a systematic, globally coherent manner (sheaf). Systematicity is afforded by a universal construction (sheaving). Sheaving infers non-local information from locally sourced knowledge to construct the nearest sheaf by gluing together data that agree on the overlapping regions (global coherency). Three examples were given: (1) inferring the ranks and suits of every card, given ranks and suits of some cards, (2) inferring the binding of features to objects given the binding of features to locations and (3) inferring object location given binocular line of sight. In each case, local knowledge is extended (composed) to infer non-local information, and this form of compositionality depends on the topology.

Note that there are two senses in which sheaving spans a formal divide. There is a ‘vertical’ sense in that presheaves are maps that preserve spatial relations (inclusions) as algebraic relations (restrictions). We limited ourselves to the simplest case where attached data were sets. In general, other categories can be used, such as categories of partially ordered sets, or groups. And there is a ‘horizontal’ sense in that data attached to open sets are glued together to construct data attached to a larger open set. These two senses arise because functors are maps between categories, whereas natural transformations (sheavings) are maps between functors.

This sheaf theory approach can be compared/contrasted with classical approaches to compositionality. Classical compositionality, in comparison, says that representations of complex entities are given by representations of their constituent entities so that the semantic relations between constituents are preserved by syntactic relations between corresponding symbolic representations. Functors preserve structure. So, classical and categorical approaches are similar to the extent that classical structures are category-like. Classical theory assumes symbolic representations are instantiated on some physical system, e.g. memory registers (or, slots), hence classical systems are sometimes called physical symbol systems [14]. Given a set of registers, one can impose the discrete topological space, in which the instantiated symbols are data attached to that space, thus realizing a presheaf. In this way, classical compositionality can be seen as an instance of categorical compositionality. By contrast, however, functoriality is only one part of the categorical approach to compositionality presented here. Presheaves and sheaves are functors, but only presheaves that are sheaves satisfy the global coherency conditions.

As noted elsewhere [17], pullbacks are reminiscent of symbolic connectionist models, LISA [21] and DORA [22]. The idea is that (relational) entities are represented via connections to corresponding neurons representing the constituent entities (fillers) and their roles in the relation based on shared semantic information represented by a common pool of neurons. Neurons representing related entities that have shared semantic features tend to bind together. Similarly, the pullback of morphisms *f* : *A* → *C* and *g* : *B* → *C* is a generalized intersection of *A* and *B* constrained by *C*. In terms of those models, objects *A* and *B* pertain to roles and fillers, *C* to semantic features, and the pullback object to relational binding. This correspondence is suggestive of a way to connect sheaving to neural network models. Neurons are topologically organized and their activities are the attached data.

The nature of sheaves depends on the nature of the data and the underlying topology. The examples of sheaves presented here are relatively simple. Sheaf theory has applications in other areas that may be adaptable to cognition. For example, a sheaf theory approach to sensor fusion [23] suggests applications to the psychology of perception. Human probability judgments that violate classical probability laws motivate quantum probability theory for cognition [24]. The close connection between sheaf theory and contextuality effects in quantum physics [25] suggests that our sheaving approach to semantic compositionality may also be applicable to quantum-like compositionality effects [17]. In these applications, the data are measurements, or probabilities [23,25].

One important direction for further work is modelling the development of the underlying topological space. Our examples illustrate how different topologies ground relational information differently. However, we have not considered how these topological spaces are obtained. Sheaf theory methods in applied topology [26] may be useful here, where the underlying topological space is inferred from data.

The importance of the underlying topology is another way that the sheaving approach goes beyond classical and artificial neural network approaches to compositionality. In this paper, we focused on the universal morphism aspect of sheaves and sheaving, because universal morphisms were argued to play a crucial role in explaining systematicity [9,11], which is a cognitive property motivating compositionality principles [8]. Yet, the topological aspect of sheaving is also crucial. Any set of registers or neurons can be given a topology. The deeper question is *why* one topology arises over another. Discrete and indiscrete topologies were asserted for an application of sheaving [17] because they are two extremes obtained from universal morphisms. So, their determination accords with the general universal construction principle [9,11]. Determination of other topologies will depend on other constraints. For instance, the physical (geometrical) relations between sensors ground triangulation of object location. This view of semantics differs from the classical view, which regards the computational (psychological) level as supported by, but independent of the specific physical (implementational) level—just as a programming language is supported by, but independent of a specific computer.

Topology captures order, and order is implicit even in the productive (recursive) aspects of cognition, e.g. level within a tree hierarchy. We have not dealt with productivity, as it purportedly implies recursion in language [27]. Category theory also provides general constructions for recursion [28], and these methods have been applied to some aspects of cognition [9]. Topology is not regarded as the only source of semantic information. So, in this sense, category (sheaf) theory provides a general framework for semantic compositionality.

## Figures and Tables

**Figure 1.**
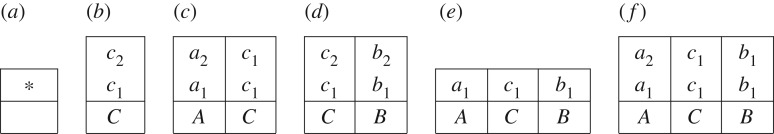
Relational tables for a presheaf (*a*–*e*) and its nearest sheaf (*a*–*d,f*).

**Figure 2.**
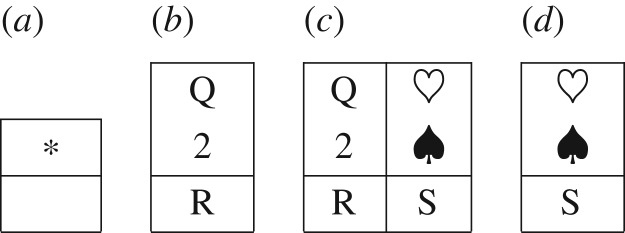
Relational tables for the presheaf $F_{Q2}$.

## Data accessibility

This article does not contain any additional data.

## Appendix A. Basic theory

Conceptual introductions to the formal concepts provided in this appendix can be found in [23,29,30], see also in [17]. Deeper introductions to the category theory concepts can be found in [13,16,19] and sheaf theory concepts in [16,18]. Specific results are referenced where they appear in the appendix.

Definition A.1 (Category). A *category* **C** consists of a collection of *objects*, O(C)={A,B,…}, a collection of *morphisms*, M(C)={f,g,…}—a morphism written in full as *f* : *A* → *B* indicates object *A* as the *domain* and object *B* as the *codomain* of *f*—including for each object A∈O(C) the *identity morphism* 1_*A*_ : *A* → *A*, and a *composition* operation, ◦, that sends each pair of *compatible* morphisms *f* : *A* → *B* and *g* : *B* → *C* (i.e. the codomain of *f* is the domain of *g*) to the *composite* morphism *g* ◦ *f* : *A* → *C*, that together satisfy the laws of:

— *identity*: *f* ◦ 1_*A*_ = *f* = 1_*B*_ ◦ *f* for every f∈M(C), and
— *associativity*: *h* ◦ (*g* ◦ *f*) = (*h* ◦ *g*) ◦ *f* for every triple of compatible morphisms f,g,h∈M(C).

Example A.2 (Set). The collection of sets (objects) and the collection of functions between sets (morphisms) form a category, denoted **Set**. The composition operation is function composition and the identity morphisms are the identity functions.

Remark A.3. Morphisms may be transformations, or relations between objects that may have there own internal structure, in which case morphisms typically preserve that structure. A category may have zero or more morphisms from an object *A* to an object *B*. In **Set**, the number of functions *A* → *B* is *n*^*m*^, where *m* (*n*) is the size of *A* (*B*). Composition need not return a ‘new’ morphism: e.g. *f* ◦ 1_*A*_ = *f* (composition with an identity); *f* ◦ *f* = *f* (self-composition of an *idempotent* function).

Definition A.4 (Topological Space). A *topological space* (*X*, *T*) consists of a set *X* and a collection *T* of subsets *U* of *X*, called the *open sets* of *T*, such that the empty set (∅) and *X* are open sets, and arbitrary unions and finite intersections of open sets are open sets. The set *T* is called the *topology* of *X*.

Example A.5 (Topological space). A topological space is a category of open sets (objects) and inclusions (morphisms)—there is just one morphism *V* → *U* if *V* ⊆ *U*. Composition is by transitivity: V⊆U∧W⊆V⇒W⊆U. The discrete topology on *X* is the set of all subsets of *X*; the indiscrete topology on *X* is {∅,X}.

Definition A.6 (Product). A *product* of objects *A* and *B*, in a category **C**, is an object *P* (also written *A* × *B*) together with a pair of morphisms *π*_1_ : *P* → *A* and *π*_2_ : *P* → *B* such that for every object *Z* and morphisms *f* : *Z* → *A* and *g* : *Z* → *B* there exists a unique morphism *u* : *Z* → *P* such that *f* = *π*_1_ ◦ *u* and *g* = *π*_2_ ◦ *u*. Morphism *u* is also denoted 〈*f*, *g*〉, as it is uniquely given by *f* and *g*.

Example A.7 (Product). A product of *A* and *B*, in **Set**, is the Cartesian product: the set *A* × *B* = {(*a*, *b*)|*a* ∈ *A*, *b* ∈ *B*} and projections $\pi_{1}\;:\;(a,b)\mapsto a$ and $\pi_{2}\;:\;(a,b)\mapsto b$.

Remark A.8. The function *u* : *Z* → *P* need not be a one-to-one correspondence (bijection). For instance, the rules of a game may stipulate that certain cards are duplicated or withheld, so a deck may contain more or less than 52 cards, i.e. the map from faces to cards, card : Face→Card, is onto (surjection) or into (injection).

Definition A.9 (Pullback). A *pullback* of morphisms *f* : *A* → *C* and *g* : *B* → *C*, in a category **C**, is an object *P* (also written *A* × _*C*_*B*) together with a pair of morphisms *π*_1_ : *P* → *A* and *π*_2_ : *P* → *B* such that for every object *Z* and morphisms *z*_1_ : *Z* → *A* and *z*_2_ : *Z* → *B* there exists a unique morphism *u* : *Z* → *P* such that diagram *commutes*: paths (where one path has at least two arrows) with the same start/end object are equal, e.g. *f* ◦ *π*_1_ = *g* ◦ *π*_2_. The dashed arrow indicates uniqueness.

Example A.10 (Pullback). A pullback of functions *f* : *A* → *C* and *g* : *B* → *C*, in **Set**, is the subset of pairs of elements {(*a*, *b*) ∈ *A* × *B*|*f*(*a*) = *g*(*b*)} and functions *π*_1_, *π*_2_.

Remark A.11. A pullback is a (generalized) product constrained by *f* and *g*. A product of *A* and *B* is equivalently a pullback of *f* : *A* → 1 and *g* : *B* → 1, where 1 is *terminal*: an object such that for every object *X*, in **C**, there exists a unique morphism from *X* to 1. In **Set**, a terminal is any singleton set, thence *f*(*a*) = *g*(*b*) for all *a* ∈ *A* and *b* ∈ *B*. Thus, a product is effectively an ‘unconstrained’ pullback.

Definition A.12 (Functor). A *functor* is a ‘structure-preserving’ map from a category **C** to a category **D**, written *F* : **C** → **D**, sending each object *A* and morphism *f* : *A* → *B* in **C** to the object *F*(*A*) and the morphism *F*(*f*) : *F*(*A*) → *F*(*B*) in **D** (respectively) that satisfies the laws of:

— *identity*: *F*(1_*A*_) = 1_*F*(*A*)_ for every object A∈O(C), and
— *compositionality*: *F*(*g* ◦ _**C**_ *f*) = *F*(*g*) ◦ _**D**_ *F*(*f*) for every pair of compatible morphisms f,g∈M(C).

Remark A.13. A functor written *F* : **C**^op^ → **D** indicates that the directions of the morphisms in **C** are reversed.

Example A.14 (Diagonal, product). The *diagonal functor* Δ : A↦(A,A),f↦(f,f) sends each object and each morphism to their pairs. The *product functor* Π : (A,B)↦A×B,(f,g)↦f×g sends pairs of objects/morphisms to their products.

Definition A.15 (Natural transformation). Let *F*, *G* : **C** → **D** be functors. A *natural transformation* $\eta \;:\;F\overset{.}{\to }G$ is a family of **D**-morphisms $\left\{\eta_{A}\;:\;F(A)\to G(A)|A\in O(C)\right\}$ such that *G*(*f*) ◦ *η*_*A*_ = *η*_*B*_ ◦ *F*(*f*) for every morphism *f* : *A* → *B* in **C**.

Example A.16 (Projection). Projection, *π* = {*π*_1_, *π*_2_}, is a natural transformation.

Definition A.17 (Universal morphism). A *universal morphism* from functor *F* : **C** → **D** to object *Y* in **D** is a pair (*B*, *ψ*) consisting of an object *B* in **C** and a morphism *ψ* : *F*(*B*) → *Y* in **D** such that for every object *X* in **C** and every morphism *g* : *F*(*X*) → *Y* in **D** there exists a unique morphism *u* : *X* → *B* in **C** such that *g* = *ψ* ◦ *F*(*u*).

Example A.18 (Products, pullbacks). A product of *A* and *B* is a universal morphism (*A* × *B*, *π*) from the diagonal functor, Δ, to the pair of objects (*A*, *B*), where *π* = (*π*_1_, *π*_2_). A pullback of morphisms *f* : *A* → *C* and *g* : *B* → *C* is a universal morphism (*A* × _*C*_*B*, *π*) from the (*generalized*) diagonal functor [10] to the pair of morphisms (*f*, *g*).

Remark A.19. Universal morphisms (e.g. products/pullbacks) are *unique up to unique isomorphism*: e.g. (*B* × *A*, *π*′), where *π*′ = (*π*_2_, *π*_1_) is also a product of *A* and *B*, but there is just one isomorphism (*A* × *B*, *π*) ≅ (*B* × *A*, *π*′) making the associated diagram commute. An isomorphism *A* × *B* ≅ *B* × *A* is generally not unique. (*f* : *X* → *Y* is an isomorphism if it has a *left/right inverse*, *g* : *Y* → *X*, i.e. *f* ◦ *g* = 1_*Y*_ and *g* ◦ *f* = 1_*X*_.)

Definition A.20 (Presheaf). Let (*X*, *T*) be a topological space. A *presheaf* is a functor $F\;:\;T^{\mathrm{op}}\to Set$ such that for each open set *U* in *T* there is a set F(U) of elements, called the *sections* over *U*, and for each inclusion *V* ⊆ *U* in *T* there is a morphism, called a *restriction morphism*, $f{|}_{V}\;:\;F(U)\to F(V)$ that satisfies:

— *identity*: for each open set *U* in *T*, the restriction morphism $f{|}_{U}\;:\;F(U)\to F(U)$ is the identity morphism $1_{F(U)}$, and
— *compositionality*: for each triple of open sets *U*, *V*, *W* in *T*, if *W* ⊆ *V* ⊆ *U*, then *g*|_*W*_ ◦ *f*|_*V*_ = (*g* ◦ *f*)|_*W*_.

Definition A.21 (Sheaf). A sheaf is a presheaf $F\;:\;T^{\mathrm{op}}\to Set$ that satisfies:

— *gluing* (existence): if {*U*_*i*_}_*i*∈*I*_ is an open cover of an open set *U* ∈ *T*, and if for each *i* ∈ *I* a section $s_{i}\in F(U_{i})$ is given such that for each pair (*U*_*i*_, *U*_*j*_) of covering sets $s_{i}{|}_{U_{i}\cap U_{j}}=s_{j}{|}_{U_{i}\cap U_{j}}$, i.e. *s*_*i*_ and *s*_*j*_ agree on overlap, then there is a section s∈F(U) such that $s{|}_{U_{i}}=s_{i}$ for each *i*, and
— *locality* (uniqueness): if {*U*_*i*_}_*i*∈*I*_ is an open cover of an open set *U* ∈ *T*, and if *s*, *t* ∈ *F*(*U*) such that $s{|}_{U_{i}}=t{|}_{U_{i}}$ for each *U*_*i*_, then *s* = *t*.

Remark A.22. The sheaf conditions are indicated by commutative diagram where the right diamond indicates the pullback of $f{|}_{U\cap V}$ and $g{|}_{U\cap V}$; or equivalently, by the *equalizer* of pairs of morphisms $\prod_{i}F(U_{i})⇉\prod_{i,j}F(U_{i}\cap U_{j})$, where the products iterate over the open sets [16].

Remark A.23. *Sheaving* [18] constructs the ‘nearest’ (smallest) sheaf $F^{+}$ from presheaf F, i.e. the universal morphism $\left(F^{+},\theta \right)$, where $\theta \;:\;F\to F^{+}$.

Example A.24 (Natural join). A perspicuous illustration of sheaving pertains to the *natural join* of relational tables [25], which involves joining tables at their shared attribute values. Attributes (headings) constitute open sets of the topological space and rows constitute the sections attached to the open sets. Restrictions are just projections along the specified attributes. Relational tables illustrate sheaving (figure 1) where table headings correspond to the open sets of the topology {∅,{C},{A,C},{B,C},{A,B,C}}. Tables (*a*–*e*) constitute a presheaf, where (*a*) is the empty table. The natural join of tables (*c*) and (*d*) constructs table (*f*), which constitutes the sheaf, i.e. tables (*a*–*d*, *f*).

Remark A.25. For the empty set, F : ∅↦1, i.e. the terminal for **Set**, written {*}, as the element’s name is unimportant. Restriction F(U)→1, for inclusion ∅⊆U, is guaranteed to exist, since 1 is terminal. The attached data is an empty function.

Example A.26 (Two cards). The *queen of hearts* and *two of spades* are represented by the presheaf $F_{Q2}$, which is expressed as a collection of relational tables (figure 2).

Example A.27 (Deck of cards). Knowing each rank and suit corresponds to a presheaf, $F_{\mathrm{card}}$, that sends the open sets {R} and {S} to the sets of all ranks and all suits, respectively. Sheaving computes the pullback of restrictions $F_{\mathrm{card}}(\{\text{R}\})\to 1$ and $F_{\mathrm{card}}(\{\text{S}\})\to 1$, since {R}∩{S}=∅, which is equivalent to the product $F_{\mathrm{card}}(\{\text{R}\})\times F_{\mathrm{card}}(\{\text{S}\})$. Thus, sheaving constructs the sheaf $F_{\mathrm{card}}^{+}$ representing the deck of all 52 cards.

Remark A.28. A topological space, (*X*, *T*), induces a specialization preorder on the elements of the underlying set, *X*. Two elements *x*, *y* ∈ *X* are comparable, *x* ≤ *y*, if *x* is an element of the *closure* of *y*, i.e. the intersection of all *closed sets* containing *y*—if *U* is an open set of *T*, then the complement of *U* (i.e. the set of elements in *X* that are not in *U*) is a closed set. In the cards example, the indiscrete topology has closed sets ∅ and {R, S}. The closure of R and the closure of S are the same set, {R, S}. Hence, the preorder has R ≤ S and S ≤ R. Open sets specify closeness. Accordingly, the open set {R, S} says that R and S are close to each other, but not preferentially so, since there are no other open sets. The open sets of a discrete topology are also the closed sets. So, in the discrete case, R and S are not comparable, since R is not in the closure of S, i.e. {S}, and S is not in the closure of R, i.e. {R}. Note that an element is always comparable to itself, *x* ≤ *x*, because any topology *T* on *X* must contain *X* as an open set of *T* (by definition).
